# Age Differences in Estimating Physical Activity by Wrist Accelerometry Using Machine Learning

**DOI:** 10.3390/s21103352

**Published:** 2021-05-12

**Authors:** Mamoun T. Mardini, Chen Bai, Amal A. Wanigatunga, Santiago Saldana, Ramon Casanova, Todd M. Manini

**Affiliations:** 1 Department of Aging and Geriatric Research, College of Medicine, University of Florida, Gainesville, FL 32610, USA; tmanini@ufl.edu; 2 Department of Health Outcomes and Biomedical Informatics, College of Medicine, University of Florida, Gainesville, FL 32610, USA; chenbai@ufl.edu; 3 Department of Epidemiology, Bloomberg School of Public Health, Johns Hopkins University, Baltimore, MD 21205, USA; awaniga1@jhu.edu; 4 Department of Biostatistics and Data Science, School of Medicine, Wake Forest University, Winston-Salem, NC 27101, USA; ssaldana@wakehealth.edu (S.S.); casanova@wakehealth.edu (R.C.)

**Keywords:** wrist, accelerometer, physical activity, energy expenditure, machine learning, random forest, age groups

## Abstract

Accelerometer-based fitness trackers and smartwatches are proliferating with incessant attention towards health tracking. Despite their growing popularity, accurately measuring hallmark measures of physical activities has yet to be accomplished in adults of all ages. In this work, we evaluated the performance of four machine learning models: decision tree, random forest, extreme gradient boosting (XGBoost) and least absolute shrinkage and selection operator (LASSO), to estimate the hallmark measures of physical activities in young (20–50 years), middle-aged (50–70 years], and older adults (70–89 years]. Our models were built to recognize physical activity types, recognize physical activity intensities, estimate energy expenditure (EE) and recognize individual physical activities using wrist-worn tri-axial accelerometer data (33 activities per participant) from a large sample of participants (*n* = 253, 62% women, aged 20–89 years old). Results showed that the machine learning models were quite accurate at recognizing physical activity type and intensity and estimating energy expenditure. However, models performed less optimally when recognizing individual physical activities. F1-Scores derived from XGBoost’s models were high for sedentary (0.955–0.973), locomotion (0.942–0.964) and lifestyle (0.913–0.949) activity types with no apparent difference across age groups. Low (0.919–0.947), light (0.813–0.828) and moderate (0.846–0.875) physical activity intensities were also recognized accurately. The root mean square error range for EE was approximately 1 equivalent of resting EE [0.835–1.009 METs]. Generally, random forest and XGBoost models outperformed other models. In conclusion, machine learning models to label physical activity types, activity intensity and energy expenditure are accurate and there are minimal differences in their performance across young, middle-aged and older adults.

## 1. Introduction

Regular and sufficient amounts of physical activity (PA) are significant in increasing health benefits and mitigating health risks. Globally, one out of four adults (almost 1.4 billion) do not meet the World Health Organization (WHO) PA recommendations [1]. Mobility is an essential factor for independence and social life engagement. Those who lose mobility have higher risk of morbidity, disability, and mortality [2,3,4,5]. Recently, WHO has published the Global action plan on physical activity 2018–2030 (GAPPA) to enhance PA with a target of 15% reduction in physical inactivity by the year 2030 [6]. The most recent WHO guidelines on physical activity and sedentary behavior [7] suggest that adults (aged 18 and older) should do at least 150–300 min of moderate-intensity aerobic PA or at least 75–150 min of vigorous intensity aerobic PA, or an equivalent combination of moderate- and vigorous-intensity activity throughout the week. Additionally, adults should replace their time spent being sedentary with PA.

To meet the WHO goals, accurate estimation of physical activity type, intensity and duration are required. The proliferation of fitness trackers and wearable accelerometers offer an excellent opportunity to achieve this goal. The literature contains many examples of machine learning algorithms used for the processing and modeling of the accelerometer data including decision tree [8], random forest [8,9], bag-of-words [10], neural network [11] and others [12,13,14,15,16,17,18,19]. However, these models are often limited to a specific age group (e.g., adults 20–40 years old). The looming question here is whether known age differences in movement patterns influence the performance of the machine learning models. There is a paucity of research to examine the differences between models built to recognize PA type and intensity, recognize individual PA, and estimate energy expenditure (EE) across different age groups. Such knowledge will be useful in deriving age-specific models that improve prediction accuracy.

Historically, the adopted approach used to recognize PA type and intensity and to estimate energy expenditure (EE) relied on data collected from the hip position in standardized laboratory settings. The advantage of the hip over other positions is the proximity to the body’s center of the mass, offering a convenient and accurate approach for capturing ambulatory activity [20]. However, the hip position is riddled with patient/participant compliance issues and inability to gather 24 h data [21]. Alternatively, the wrist position has become popular for collecting accelerometer data due to a rise in smartwatches, convenience, ability to capture sleep quality (24 h) and enhanced compliance in research studies [22,23,24,25]. Unfortunately, despite the popularity of wrist-worn accelerometers, there is a paucity of models that are deemed viable for accurately assessing PA [26,27]. The use of the wrist position to recognize PA type and intensity and estimate EE is challenging due to its potential limitation in quantifying and capturing large lower limb movements and other lifestyle activities. Therefore, models that can accurately recognize PA type and intensity and estimate energy expenditure from the wrist are greatly needed to meet the current demand.

**Figure 1:**
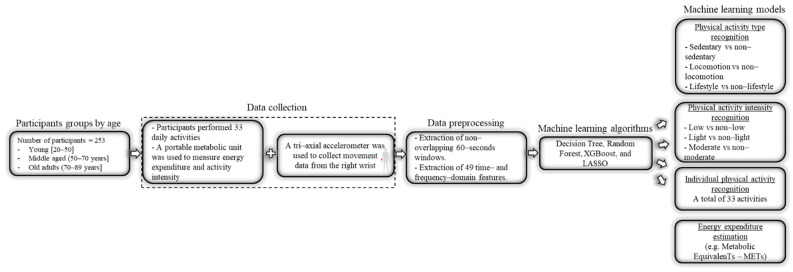
A block diagram showing the steps followed to collect and process the data.

This study utilizes a large amount of high-resolution raw accelerometer data collected from the wrist position coupled with metabolic intensity assessed in 253 adults aged 20–89 years. An aggregated set of relevant features was used as an input to machine learning models to recognize PA type and intensity, identify individual PA, and estimate EE. Machine learning models developed on specific age groups (young [20–50], middle (50–70] and old (70–89]) were then compared to test the hypothesis that model performance varies across age group, as shown in Figure 1. Results are expected to help evaluate whether machine learning models used to represent wrist-worn accelerometer data need to be tailored to known age differences in movement and behavior to optimize their accuracy.

## 2. Materials and Methods

### 2.1. Participants

Participants were community dwelling adults 20+ years old who were able to read and speak English language, were willing to undergo all testing procedures, and their weight was stable in the last three months (+/−5 lbs). Two-hundred and fifty-three (253) of the 264 participants who were enrolled were included in the analysis. Those excluded either had: missing start/end time of activities (6 participants), insufficient length of activity or missing values (3 participants) and missing demographic information (2 participants). The Institutional Review Board at the University of Florida approved all study procedures, and all participants provided written informed consents before the study.

### 2.2. Prescribed Activities and Visits

The ChoresXL study methods have been described previously by our group [28,29]. Briefly, participants performed a battery of 33 typical daily activities that were categorized into activity types and intensities calculated post-facto from metabolic unit data (Supplemental Table S1). Tasks were chosen because they mimic daily chores activities, common among most Americans, and they are consistent with average time spent in the 2010 American Time Use Survey [30]. All tasks were performed in a standardized laboratory setting with scripted instructions for approximately 8–10 min to achieve a steady state energy expenditure. Participants performed all tasks at their own speed and were ordered from lowest to highest metabolic demand to reduce transfer of high metabolic effects of one task to another. To ease burden and exhaustion, participants performed all tasks over four visits. However, some did not complete all visits. Overall, 213 participants attended all 4 visits, 21 attended 3 visits, 7 attended only 2 visits, and 12 attended only 1 visit. In total, there were 941 data collection visits.

### 2.3. Instrumentation

Participants wore ActiGraph GT3X-BT monitors on their right wrists (ActiGraph Inc, Pensacola, FL, USA). The ActiGraph GT3X-BT monitor is a tri-axial lightweight accelerometer that records accelerations in units of gravity (1 g) in perpendicular, anterior-posterior and medio-lateral axes. Accelerometers were programmed to collect data at 100 Hz sampling rate. Participants also wore a 2 kg portable metabolic unit that estimated energy expenditure using principles of indirect calorimetry, Cosmed K5 (COSMED, Rome, Italy). Before data collection, the oxygen (O_2_) and carbon dioxide (CO_2_) sensors were calibrated using a gas mixture sample of 16.0% O_2_ and 5.0% CO_2_ and room air calibration. The turbine flow meter was calibrated using a 3.0-L syringe. A flexible facemask was positioned over the participant’s mouth and nose and attached to the flow meter. Oxygen consumption (VO_2_ = mL·min^−1^·kg^−1^) was measured breath-by-breath and was subsequently smoothed with a 30-s running average window. Steady-state VO_2_ for each task was manually calculated over approximately 2 min when there was evidence of a plateau, which indicates metabolic demand is matched to physical workload. Data were expressed as METs after dividing the VO_2_ values by the traditional standard of 3.5 mL·min^−1^·kg^−1^ [31].

### 2.4. Problem Formulation

In this paper, we targeted four main tasks to measure the hallmark measures of PA: (1) recognize PA type (classification task) through splitting this task into three binary classification tasks: (i) sedentary vs. non-sedentary; (ii) locomotion vs. non-locomotion and iii) lifestyle vs. non-lifestyle; (2) recognize PA intensity (classification task) through splitting this task into three binary classification tasks: (i) low vs. non-low; (ii) light vs. non-light and (iii) moderate vs. non-moderate; (3) recognize individual PA (classification task) and (4) estimate the energy expenditure while performing the scripted activities (regression task). We extracted consecutive non-overlapping 60-s windows from the raw accelerometer data. Previous studies used various window lengths, ranging from 0.1 s to 128 s [32,33,34,35]. A 60-s window was chosen as a compromise between having sufficient data for accurate feature extraction and balancing computational resources. In total, 49 time—and frequency—domain features, listed in Table 1, were extracted. Although there is inconsistency among researchers about the aggregation of relevant features, in this work, we combined features from previous studies [8,36,37,38,39,40]. During data processing, some cases with different collection frequencies were discovered (15 at 80 Hz and 100 at 30 Hz). However, no resampling was performed because the resolution was sufficient to extract features over a 60-s window.

### 2.5. Model Training

Four main models were developed for PA type recognition, PA intensity recognition, EE estimation and individual PA recognition. The models were generated separately across three age groups: young [20–50 years], middle (50–70 years], and old (70–89 years]. For EE estimation, 247 participants provided valid data and were included. All the scripted activities (33 activities) were used in the case of individual PA recognition, PA intensity recognition and EE estimation. However, for PA type recognition, some activities were removed (strength exercise leg extension, strength exercise chest press, strength exercise leg curl, stretching yoga); they did not fit sedentary, locomotion or lifestyle categories. We utilized four algorithms to build our machine learning models: decision tree [40], random forest [40], extreme gradient boosting (XGBoost) [41] and least absolutes and selection operator (LASSO) [42]. Our selection of these algorithms was based on the fact that all the models provide better interpretability and include feature selection as part of the model building process. For the PA type recognition, we built binary classification models for each type and age group, resulting in 48 models. Similarly, for the PA intensity recognition, we built binary classification models for each intensity and age group, resulting in 48 models. For individual PA recognition, we built one multi-class classification model (33 classes) for each age group using the best performing model (XGBoost), resulting in 4 models. For EE estimation, we built one regression model for each group, resulting in 16 models. All the utilized machine learning algorithms are naturally resistant to insignificant predictors. They intrinsically perform feature selection to enhance the predictability of the models [43]. In all tasks, all participants were randomly distributed into 5 folds. In order to solve the data imbalance, the models were set to automatically adjust weights inversely proportional to the frequencies of the classes in the input data. We used 5-fold nested cross validation (nested-CV), which uses a series of train, validation and test set splits. The nested CV consists of an inner CV loop nested in an outer CV loop. The inner loop is responsible for hyperparameter tuning (the process of searching for the optimal parameters of the model), while the outer loop is responsible for error estimation and generalization. Initially, the data is split into outer training and testing datasets (outer loop). Then, the outer training dataset is further split into inner training and testing datasets (inner loop). Validation and hyperparameter tuning happen in the inner datasets, then the performance is reported on the outer testing datasets. This process was repeated 5 times. Then, the model with the highest performance was chosen. In this approach, the model selection becomes an integral part of the model fitting process, which results in preventing bias in performance evaluation [44,45,46]. We used four metrics to report the models’ performance: F1-Score = 2 × (precision × recall)/(precision + recall), area under the curve (AUC) = area under true positive rate vs. false positive rate, balanced accuracy = (sensitivity + specificity)/2 and accuracy. The F1-Score measures the harmonic mean of precision and recall. The F1-Score was used to compare across age groups, because it protects against the imbalance across classes seen in PA type and intensity categories. There is no absolute criterion for a “good” value of F1 measure, but values above 0.80 generally indicate good performance. For the continuous data from energy expenditure (METs), the root mean square error (RMSE) was used to evaluate performance.

### 2.6. Brief Overview of the Utilized Machine Learning Algorithms

LASSO (least absolute shrinkage and selection operator) is a statistical and machine learning regression algorithm used for feature selection and regularization to enhance the performance of the model. It is a type of linear regression that utilizes the concept of shrinkage, in which the data points are shrunk towards a central point [47]. We utilized the *Glmnet* package [47], in which the LASSO linear and logistic regression are implemented.

Decision tree learning is one of the most common machine learning algorithms due to its simplicity and interpretability. The tree is a graphical representation of decisions. It consists of leaves representing the class labels (e.g., sedentary or non-sedentary), and branches representing conjunctions of features (e.g., time- and frequency-domain features) that lead to those class labels. The tree is built by splitting the source dataset into subsets. Each subset is used to select the feature that best split the data equally. Decision tree learning can be used for building classification trees (e.g., PA type and intensity recognition) or regression trees (e.g., EE estimation) [48].

Random forest is an ensemble learning algorithm based on the concept of bagging (or bootstrap aggregation), in which predictions from multiple decision trees are combined through a majority voting mechanism. In random forest, however, only a subset of features are selected randomly to build a forest of decision trees [40].

XGBoost (extreme gradient boosting) is also an ensemble learning algorithm based on the gradient boosting framework, in which models are built sequentially to boost (increase) the performance of the previous models by utilizing the gradient descent algorithm to minimize errors. However, XGBoost offers better hardware and software optimization mechanisms, prevents overfitting by penalizing complex models and handles sparse patterns and missing data efficiently [41].

## 3. Results

**Figure 2:**
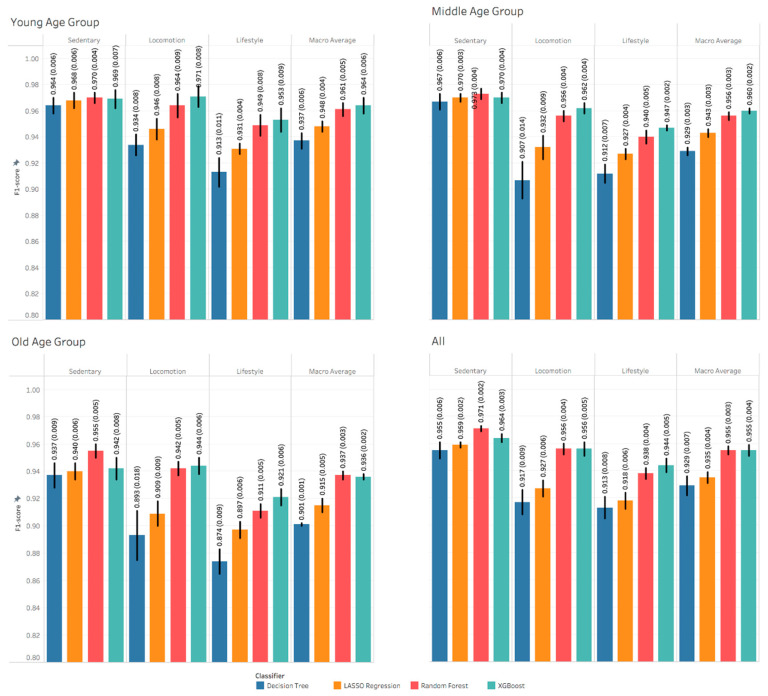
The F1-Scores of recognizing physical activity type. Each value is the mean and standard deviation of the 5-fold nested cross validation.

Table 2 shows participants’ descriptive characteristics per age group: young (20–50 years), middle (50–70 years) and old (70–89 years). There are no noticeable differences between the different age groups with respect to BMI, women percentage and number of Hispanic. Figure 2 shows a slight performance reduction from younger to older age groups and from sedentary to more high variability lifestyle activities. The performance of the machine learning models were similar in recognizing sedentary PA type and varied in recognizing locomotion and lifestyle PA types. Generally, XGBoost and random forest models outperformed other models. However, the XGBoost models were slightly better than the random forest models in most of the tasks, except for recognizing sedentary PA type across all age groups. Figures S1–S3 show other performance metrics including AUC, balanced accuracy and accuracy.

**Figure 3:**
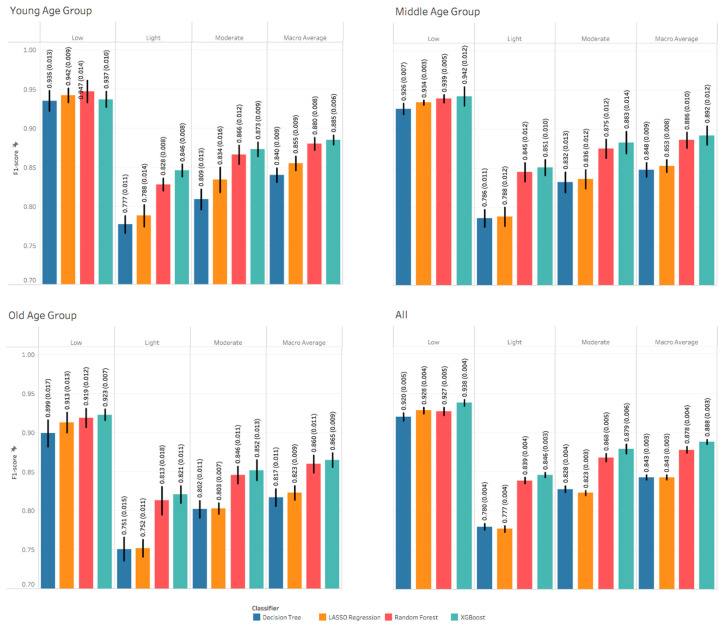
Performance metrics of recognizing physical activity intensity. Each value is the mean and standard deviation of the 5-fold nested cross validation.

Results for PA intensity show that the models’ performance was slightly higher for young and middle age groups compared to the old age group, as shown in Figure 3. The performance of low intensity models across age groups outperformed the performance of the moderate, then light intensities. The performance of the machine learning models were close in recognizing low PA intensity and varied in recognizing light and moderate PA intensities. Generally, XGBoost and random forest models outperformed other models. However, the XGBoost models were slightly better than the random forest models in most of the tasks, except for recognizing low PA intensity in the young age group. Figures S4–S6 show other performance metrics including AUC, balanced accuracy and accuracy.

**Figure 4:**
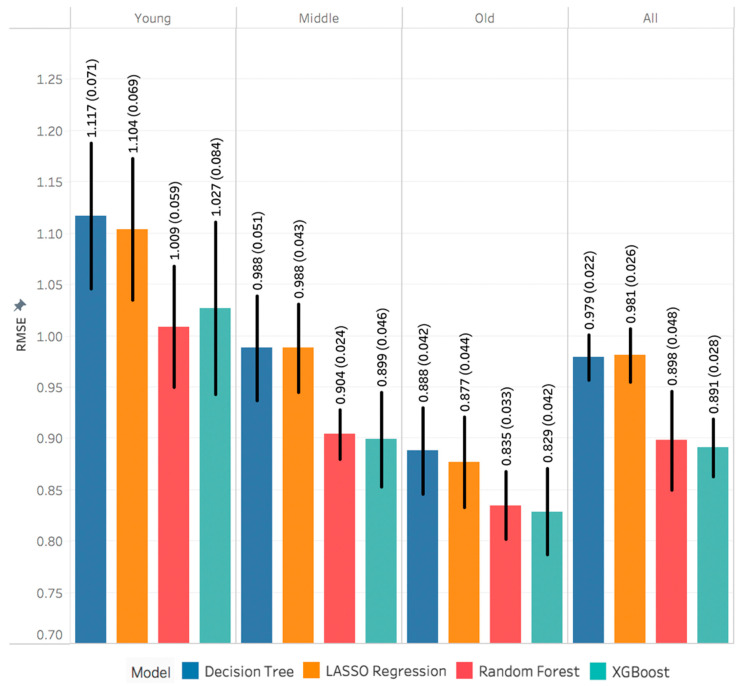
Performance metrics of estimating energy expenditure. Each value is the mean and standard deviation of the 5-fold nested cross validation.

Figure 4 shows that METs RMSE decreased (improved) from young to middle to older age groups. The performance of the machine learning models was close for the young age group and varied for the middle and old age groups. Generally, XGBoost and random forest models outperformed other models. However, the XGBoost models were slightly better than the random forest models, except for the young age group.

Table 3 shows the performance of recognizing individual PA using XGBoost. It can be noticed that activities mainly involving wrist movements (washing dishes, computer work, cleaning windows) tend to perform better than others. However, there is no clear difference across age groups.

Figures S7–S9 show the confusion matrices of recognizing PA type across age groups. The confusion increases as we move from sedentary to lifestyle PA type, which is consistent with the F1 scores shown in Figure 2. Figures S10–S12 show the confusion matrices of recognizing PA intensity across age groups. Similarly, the confusion of the models are consistent with the F1 scores shown in Figure 4.

Figures S13–S15 show the top 15 features that contributed the most in recognizing PA type across age groups extracted from the XGBoost models. It can be noticed that the ranking of features is similar across age groups within each PA type. Figures S16–S18 show the top 15 features that contributed the most in recognizing PA intensity across age groups. Similarly, it can be noticed that the ranking of features is similar across age groups within each PA intensity.

## 4. Discussion

The goal of the study was to build accurate machine learning models to recognize the hallmark measures of physical activities and estimate energy expenditure across different age groups. We analyzed a large dataset of raw accelerometer data collected from the wrist position. We utilized four machine learning algorithms to build our models including: decision tree, random forest, extreme gradient boosting (XGBoost) and least absolute shrinkage and selection operator (LASSO). Results showed that the machine learning models were quite accurate at recognizing physical activity type and intensity and estimating energy expenditure. However, models performed less optimally when recognizing individual physical activities. Our hypothesis that increasing age would impact model performance was rejected as only slight differences were detected among age groups.

The results of the models built to recognize physical activity type showed high performance for all age groups, as shown in Figure 2. Although the results were similar across age groups, there was a slightly higher performance in the young, followed by the middle, then the old age group for a majority of the activity types. Additionally, the highest performance was for sedentary, locomotion, then lifestyle activities for all age groups. Physical activity types seem to be more distinguishable and cause less confusion for younger ages as reflected on the confusion matrices shown in Figures S7–S9. It is hard to interpret the drop in the performance from young to old age groups. One potential cause of this drop is the deviations from the standardized protocol that are more common in older adults. For example, there was a certain amount of variability in the *trash removal* activity among older adults compared to younger adults (older adults could not pull the trash bag quickly). This suggests that the ML models need to incorporate these compensations more accurately among older populations. Another reason is that older adults do not like the wrist device as tight as the younger adults. This can result in unintended artifactual movement, which occurred more commonly among the older. An additional cause could be that the middle and old age groups include more participant data than the young age group. Therefore, the models tend to generalize better and be less optimistic. On the other hand, the drop in the performance from sedentary to lifestyle activity types is intuitive. Lifestyle activities typically require more wrist involvement (i.e., ironing, trash removal) than other physical activity types. This means more variability in physical activities as we move from sedentary to lifestyle activities, which can increase the confusion in recognizing physical activity types, as reflected in the confusion matrices shown in Figures S7–S9.

The results of the models built to recognize physical activity intensity showed relatively high performance for all age groups, but lower than the performance of recognizing physical activity types, as shown in Figure 3. The highest performance was for the young and middle age groups alternatively and then the old age group for all activity intensities. Additionally, the highest performance was for low, moderate, then light intensities for all age groups. As mentioned above, it is hard to interpret the drop in the performance from young to old age groups. Performance metrics and confusion for labeling physical activity intensities showed a consistent, although slight, reduction in older aged groups (see Figure 3 and Figures S10–S12). If this error was scaled to free-living conditions over a typical day (16 h), older adults would be expected to have 2% (~19 min) more mislabeling of PA intensity compared to a younger group.

Models built to recognize individual physical activities showed lower performance than recognizing physical activity type. The highest F1-Score was 0.8 for recognizing computer work activity in the middle age group and the lowest was 0.318 for recognizing walking at RPE 1 activity in the old age group. The overall deterioration in the recognition performance in individual activities compared to other recognition tasks is intuitive, due to the high number of classes and the data imbalance. Summing these activities into categories such as the physical activity types or physical activity intensities can help in enhancing the recognition performance metric, as observed in Figure 2 and Figure 3. In general, there were no consistent differences among age groups.

The scaled impurity-based feature importance ranking generated from the XGBoost algorithm show how relevant these features are to the problem in hand and help in better understanding the model. We listed the top 15 features out of 49 features for both the physical activity type and intensity recognition tasks generated from the XGBoost models. By examining the feature importance for the physical activity types, there is a consistency in the ranking of these features across age groups within each one of the activity types. For example, variability in vector magnitude features such as *sdvm* and *cv_vm* were important in predicting sedentary physical activities, whereas wrist-specific features such as *wrist_sd_z* and *sd_angle* are more relevant for recognizing lifestyle activity types. The feature importance rankings for low intensity activities were similar to sedentary PA type, where the VM features such as *sdvm* and *cv_vm* were dominant. Feature rankings for predicting light and moderate intensities were similar with high importance for moment-based variables. Similarly, there is a consistency in the feature importance ranking across the age groups suggesting that the features are robust regarding potential movement difference with increasing age. Interestingly, the amplitude of the accelerometer axis (i.e., mean VM), which is commonly used to gauge intensity did not have a major role in model prediction. Being aware of the important features for the recognition problem in hand can help researchers continue improving model accuracy with less computational costs.

Comparing relevant literature results is an intricate endeavor, because of the differences in the data collection environment and the variables that govern the study. There are numerous differences between studies, which include: sample size, the demographic characteristics of participants, the number and diversity of the physical activities tested, type of accelerometer, body position, statistical and machine learning algorithms applied, the extracted statistical features, the window size and the metrics measured to evaluate the overall performance. However, some important comparisons can be made. For example, Ellis et al. [49] built random forest models on data collected from the dominant wrist to predict physical activity type and estimate energy expenditure. The models were developed and tested on 40 (average age 35.8 years) participants. They obtained an average F1 score of 0.75 on 8 daily activities. Additionally, they obtained an RMSE value of 1.0 METs, which is similar to our young age group. Staudenmayer et al. [8] also used random forest to estimate energy expenditure and metabolic intensity of 19 physical activities from wrist accelerometer data. The models derived from a small young sample of 20 (24.1 years) estimated RMSE at 1.21 METs. When compared to others using machine learning approaches, the results from the current work are comparable within the young age group, but better in middle and old age groups. Despite the differences mentioned above, we compared our work with others who processed data collected from a triaxial accelerometer placed on the wrist position to recognize physical activity type, recognize individual activities or estimate energy expenditure, as shown in the Supplemental Table S2.

Studies that examined the hallmark measures of physical activity have used publicly available data that contain activity labels, but not measures of metabolic intensity or energy expenditure (e.g., Opportunity (multiple body positions, 3 participants) [50], PAMAP2 (chest, arm and ankle positions, 9 participants) [51], UCI daily and sports dataset (hip position, 30 participants) [52], Skoda Mini Checkpoint (multiple body positions, 1 participant) [53], WISDM (hip position, 29 participants) [54], and Daphnet Freezing of Gait Dataset (legs and hip positions, 10 participants) [52]). They are also limited by a small number of participants, the age range being mostly <40 years, a low number and diversity of activity types and, most importantly, lacking sufficient data from the wrist position. Given these substantial differences, the models presented here show relatively higher performance than others. Additionally, the current models may generalize better due to the high diversity of activities, wide age span, gender and racial diversity and the larger number of participants enrolled.

A limitation of the current study is that data were collected in controlled lab settings, which is appropriate and a first step in evaluating positional differences [55]. Collecting data in free-living settings is more reflective of the numerous transitions between activity types, but it is challenged by labeling the activity type. Another limitation is the consideration of window size, which was based on previous studies that extracted time- and frequency-domain features. This window size may not reflect the most appropriate size for all tasks and age groups. Additional simulation work should evaluate different window sizes for optimizing performance.

## 5. Conclusions

In this study, we tested the hypothesis that the performance of machine learning models in estimating activity types, activity intensity and energy expenditure would vary across age groups. Overall results suggest data features derived from wrist worn accelerometers and analyzed using machine learning models lead to high-to-moderate accuracy across all age groups. In conclusion, a generalized approach to processing wrist accelerometry data, without consideration of a person’s age, is sufficient for estimating physical activity.

## Figures and Tables

**Table 1:** Description of features extracted from the raw data.

	Feature	Description
**Time**	Mean of vector magnitude (mvm)	Sample mean of the VM in the window
	SD of vector magnitude (sdvm)	Standard deviation of VM
	Mean angle of acceleration relative to vertical on the device (mangle)	Sample mean of the angle between x axis and VM in the window
	SD of the angle of acceleration relative to vertical on the device (sdangle)	Sample standard deviation of the angles in the window
	Mean of acceleration (mean_x, mean_y and mean_z)	Sample mean of acceleration from x axis, y axis and z axis in the window
	SD of acceleration (sd_x, sd_y and sd_z)	Standard deviation of acceleration from x axis, y axis and z axis in the window
	Coefficient of variation of acceleration (cv_x, cv_y and cv_z)	Standard deviation of acceleration from x axis, y axis and z axis in the window divided by their mean, multiplied by 100
	Min of vector magnitude and acceleration (min_vm, min_x, min_y and min_z)	Min value of VM and acceleration from x axis, y axis and z axis in the window
	Max of vector magnitude and acceleration (max_vm, max_x, max_y and max_z)	Max value of VM and acceleration from x axis, y axis and z axis in the window
	25% quantile of vector magnitude and acceleration (lower_vm_25, lower_x_25, lower_y_25 and lower_z_25)	Lower 25% quantile of VM and acceleration from x axis, y axis and z axis in the window
	75% quantile of vector magnitude and acceleration (upper_vm_75, upper_x_75, upper_y_75 and upper_z_75)	Upper 75% quantile of VM and acceleration from x axis, y axis and z axis in the window
	Third moment of vector magnitude and acceleration (third_moment_vm, third_moment_x, third_moment_y and third_moment_z)	Third moment of VM and acceleration from x axis, y axis and z axis in the window, which are used to depict the shape of the signals
	Fourth moment of vector magnitude and acceleration (fourth_moment_vm, fourth_moment_x, fourth_moment_y and fourth_moment_z)	Fourth moment of VM and acceleration from x axis, y axis and z axis in the window, which are used to depict the shape of the signals
	Skewness	Skewness of the VM, acceleration from x axis, y axis, and z axis in the window
	Kurtosis	Kurtosis of the VM, acceleration from x axis, y axis and z axis in the window
	Coefficient of variation (CV)	Standard deviation of VM in the window divided by the mean, multiplied by 100
**Frequency**	Percentage of the power of the vm that is in 0.6–2.5 Hz (p625)	Sum of moduli corresponding to frequency in this range divided by sum of moduli of all frequencies
	Dominant frequency of vm (df)	Frequency corresponding to the largest modulus
	Fraction of power in vm at dominant frequency (fpdf)	Modulus of the dominant frequency/sum of moduli at each frequency

**Table 2:** Participants descriptive characteristics by age group.

	Young	Middle	Old	All
Age range, years	[20–50]	(50–70]	(70–89]	[20–89]
Mean Age (SD), years	35.2 (10.7)	61.9 (5.6)	77.7 (5.1)	61.7 (17.7)
Mean BMI (SD), kg/m^2^	26.1 (5.5)	26.9 (5.5)	27.7 (5.8)	27 (5.6)
Women %	60%	67%	58%	62%
Number of Hispanic	3	2	1	6
Total number	60	95	98	253

**Table 3:** Performance metrics of recognizing individual physical activities using XGBoost. Each value is the mean and standard deviation of the 5-fold nested cross validation.

	Young	Middle	Old	All
Individual Activities Recognition Performance (F1 Score)				
leisure walk	0.544 (0.055)	0.491 (0.070)	0.391 (0.059)	0.497 (0.026)
rapid walk	0.645 (0.055)	0.545 (0.061)	0.470 (0.048)	0.567 (0.037)
light gardening	0.585 (0.056)	0.529 (0.051)	0.495 (0.025)	0.571 (0.047)
yard work	0.416 (0.035)	0.478 (0.046)	0.404 (0.070)	0.489 (0.040)
prepare serve meal	0.520 (0.022)	0.482 (0.037)	0.480 (0.046)	0.520 (0.027)
digging	0.711 (0.040)	0.686 (0.050)	0.637 (0.053)	0.719 (0.038)
straightening up dusting	0.460 (0.051)	0.427 (0.041)	0.415 (0.027)	0.483 (0.014)
washing dishes	0.782 (0.012)	0.706 (0.024)	0.596 (0.035)	0.716 (0.023)
unloading storing dishes	0.666 (0.031)	0.669 (0.044)	0.597 (0.036)	0.675 (0.021)
walking at rpe 1	0.366 (0.064)	0.491 (0.027)	0.318 (0.056)	0.437 (0.027)
personal care	0.660 (0.043)	0.709 (0.028)	0.552 (0.027)	0.672 (0.011)
dressing	0.494 (0.035)	0.450 (0.038)	0.335 (0.023)	0.456 (0.021)
walking at rpe 5	0.482 (0.050)	0.440 (0.104)	0.356 (0.094)	0.443 (0.029)
sweeping	0.602 (0.068)	0.634 (0.073)	0.518 (0.057)	0.625 (0.018)
vacuuming	0.637 (0.029)	0.611 (0.044)	0.533 (0.035)	0.625 (0.024)
stair descent	0.705 (0.120)	0.693 (0.055)	0.635 (0.064)	0.706 (0.040)
stair ascent	0.543 (0.104)	0.561 (0.085)	0.518 (0.023)	0.600 (0.047)
trash removal	0.425 (0.047)	0.473 (0.050)	0.355 (0.017)	0.465 (0.034)
replacing sheets on a bed	0.626 (0.064)	0.677 (0.029)	0.559 (0.024)	0.665 (0.031)
stretching yoga	0.628 (0.026)	0.642 (0.033)	0.557 (0.043)	0.630 (0.035)
mopping	0.673 (0.039)	0.660 (0.033)	0.623 (0.041)	0.702 (0.041)
light home maintenance	0.507 (0.027)	0.536 (0.035)	0.459 (0.028)	0.530 (0.025)
computer work	0.780 (0.043)	0.800 (0.039)	0.759 (0.049)	0.795 (0.017)
heavy lifting	0.650 (0.031)	0.672 (0.024)	0.495 (0.041)	0.647 (0.035)
shopping	0.506 (0.052)	0.537 (0.039)	0.524 (0.033)	0.563 (0.040)
ironing	0.636 (0.033)	0.687 (0.014)	0.620 (0.056)	0.700 (0.023)
laundry washing	0.426 (0.036)	0.509 (0.039)	0.411 (0.040)	0.479 (0.021)
strength exercise leg curl	0.576 (0.044)	0.644 (0.062)	0.656 (0.108)	0.695 (0.028)
strength exercise chest press	0.681 (0.082)	0.668 (0.063)	0.602 (0.079)	0.697 (0.017)
strength exercise leg extension	0.367 (0.128)	0.462 (0.092)	0.329 (0.079)	0.419 (0.019)
tv watching	0.614 (0.050)	0.616 (0.019)	0.546 (0.069)	0.624 (0.030)
standing still	0.631 (0.081)	0.644 (0.060)	0.527 (0.094)	0.612 (0.036)
washing windows	0.764 (0.058)	0.720 (0.045)	0.739 (0.056)	0.755 (0.024)
Macro average (F1 score)	0.584 (0.023)	0.594 (0.021)	0.516 (0.011)	0.600 (0.014)

## Supplementary Materials

References [56,57] are cited in the supplementary materials. The following are available online at https://www.mdpi.com/article/10.3390/s21103352/s1, Figure S1: The receiver operating characteristic–area under the curve of recognizing physical activity type. Each value is the mean and standard deviation of the 5-fold nested cross validation, Figure S2: The balanced accuracy of recognizing physical activity type. Each value is the mean and standard deviation of the 5-fold nested cross validation, Figure S3: The accuracy of recognizing physical activity type. Each value is the mean and standard deviation of the 5-fold nested cross validation, Figure S4: The receiver operating characteristic–area under the curve of recognizing physical activity intensity. Each value is the mean and standard deviation of the 5-fold nested cross validation, Figure S5: The balanced accuracy of recognizing physical activity intensity. Each value is the mean and standard deviation of the 5-fold nested cross validation, Figure S6: The accuracy of recognizing physical activity intensity. Each value is the mean and standard deviation of the 5-fold nested cross validation, Figure S7: Confusion matrix of recognizing physical activity type for young age group, Figure S8: Confusion matrix of recognizing physical activity type for middle age group, Figure S9: Confusion matrix of recognizing physical activity type for old age group, Figure S10: Confusion matrix of recognizing physical activity intensity for young age group, Figure S11: Confusion matrix of recognizing physical activity intensity for middle age group, Figure S12: Confusion matrix of recognizing physical activity intensity for old age group, Figure S13: Feature importance for recognizing sedentary activities across age groups, Figure S14: Feature importance for recognizing locomotion activities across age groups, Figure S15: Feature importance for recognizing lifestyle activities across age groups, Figure S16: Feature importance for recognizing low intensity across age groups, Figure S17: Feature importance for recognizing light intensity across age groups, Figure S18: Feature importance for recognizing moderate intensity across age groups, Table S1: List of the performed physical activities, their type, and intensity, Table S2: Comparison with relevant work in the literature. The listed studies collected data from the wrist position for physical activity type classification. Classification performance is accuracy unless otherwise mentioned. For the purpose of comparison, we calculated the average accuracy for the physical activity type classification. N is the number of participants; Num is number; ML is machine learning; RMSE is the root mean square error; RF is random forest; SVM is support vector machine; and RLR is regularized logistic regression.
