# Supplementary material for: Age Differences in Estimating Physical Activity by Wrist Accelerometry Using Machine Learning
Source: Sensors (Basel). 2021 May 12;21(10):3352. doi: 10.3390/s21103352 (PMC8150764; doi:10.3390/s21103352)
Supplement: Supplementary file 1 [file sensors-21-03352-s001.zip › sensors-1159488-supplementary.pdf]

sensors

MDPI

Table S1. List of the performed physical activities, their type, and intensity.

	Activity Type			Intensity		
**Activity**	**Sedentary**	**Locomotion**	**Life-Style**	**Low**	**Light**	**Moderate**
leisure walk	No	Yes	No	No	No	Yes
rapid walk	No	Yes	No	No	No	Yes
light gardening	No	No	Yes	No	No	Yes
yard work	No	No	Yes	No	No	Yes
prepare serve meal	No	No	Yes	No	Yes	No
digging	No	No	Yes	No	No	Yes
straightening up dusting	No	No	Yes	No	No	Yes
washing dishes	No	No	Yes	No	Yes	No
unloading storing dishes	No	No	Yes	No	Yes	No
walking at rpe 1	No	Yes	No	No	No	Yes
personal care	No	No	Yes	No	Yes	No
dressing	No	No	Yes	No	Yes	No
walking at rpe 5	No	Yes	No	No	No	Yes
sweeping	No	No	Yes	No	No	Yes
vacuuming	No	No	Yes	No	No	Yes
stair descent	No	Yes	No	No	No	Yes
stair ascent	No	Yes	No	No	No	Yes
trash removal	No	No	Yes	No	No	Yes
replacing sheets on a bed	No	No	Yes	No	No	Yes
stretching yoga *	No	No	No	No	Yes	No
mopping	No	No	Yes	No	No	Yes
light home maintenance	No	No	Yes	No	No	Yes
computer work	Yes	No	No	Yes	No	No
heavy lifting	No	No	Yes	No	No	Yes
shopping	No	No	Yes	No	Yes	No
ironing	No	No	Yes	No	Yes	No
laundry washing	No	No	Yes	No	Yes	No
strength exercise leg curl *	No	No	No	No	Yes	No
strength exercise chest press *	No	No	No	No	Yes	No
strength exercise leg extension *	No	No	No	No	Yes	No
tv watching	Yes	No	No	Yes	No	No
standing still	Yes	No	No	Yes	No	No
washing windows	No	No	Yes	No	No	Yes

A total of 29 activities were considered for PA type recognition, 33 for individual PA recognition, PA intensity recognition, and EE estimation. * Only considered for energy expenditure estimation, PA intensity recognition, and individual PA recognition.

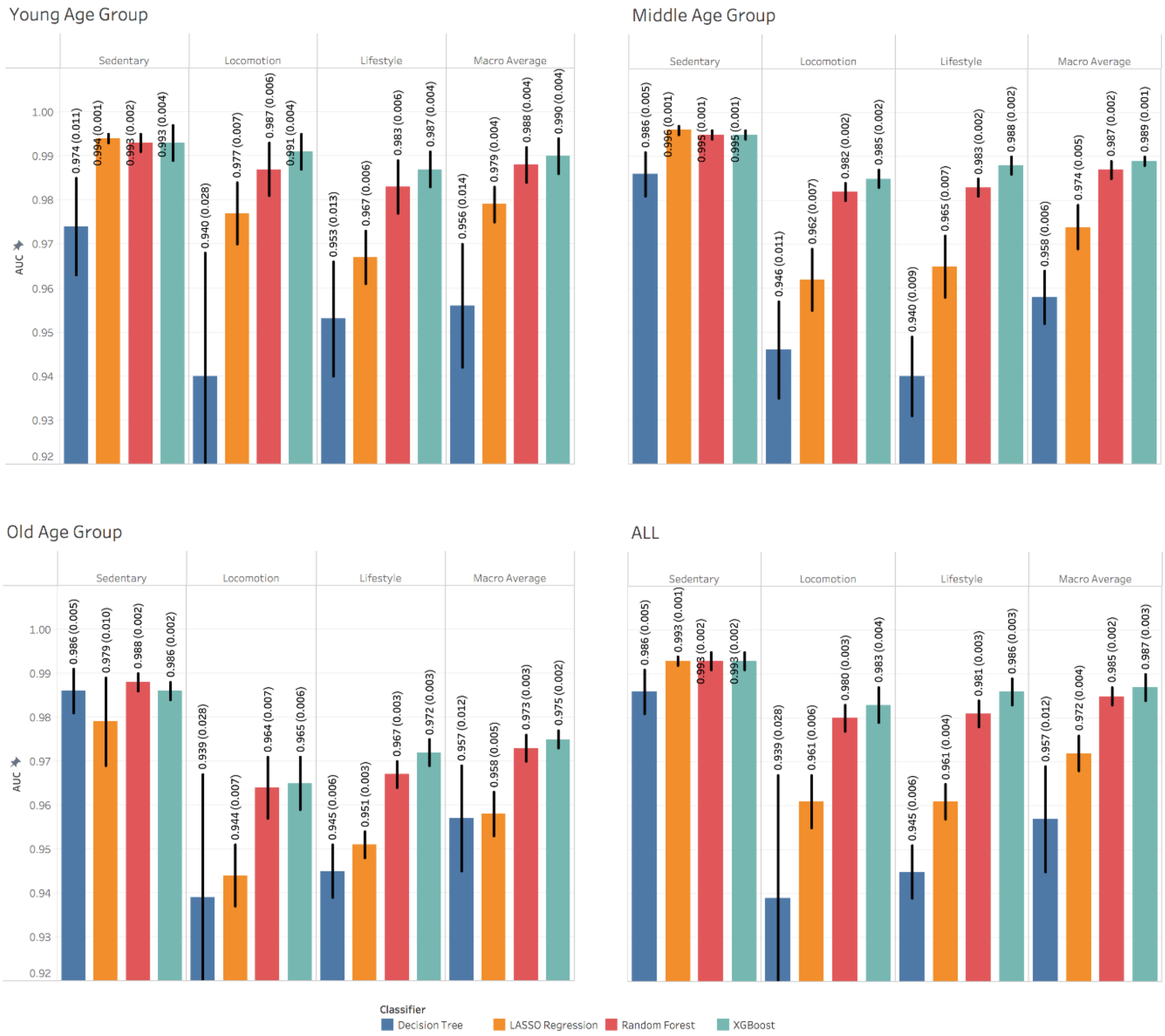

Figure S1. The receiver operating characteristic–area under the curve of recognizing physical activity type. Each value is the mean and standard deviation of the 5-fold nested cross validation.

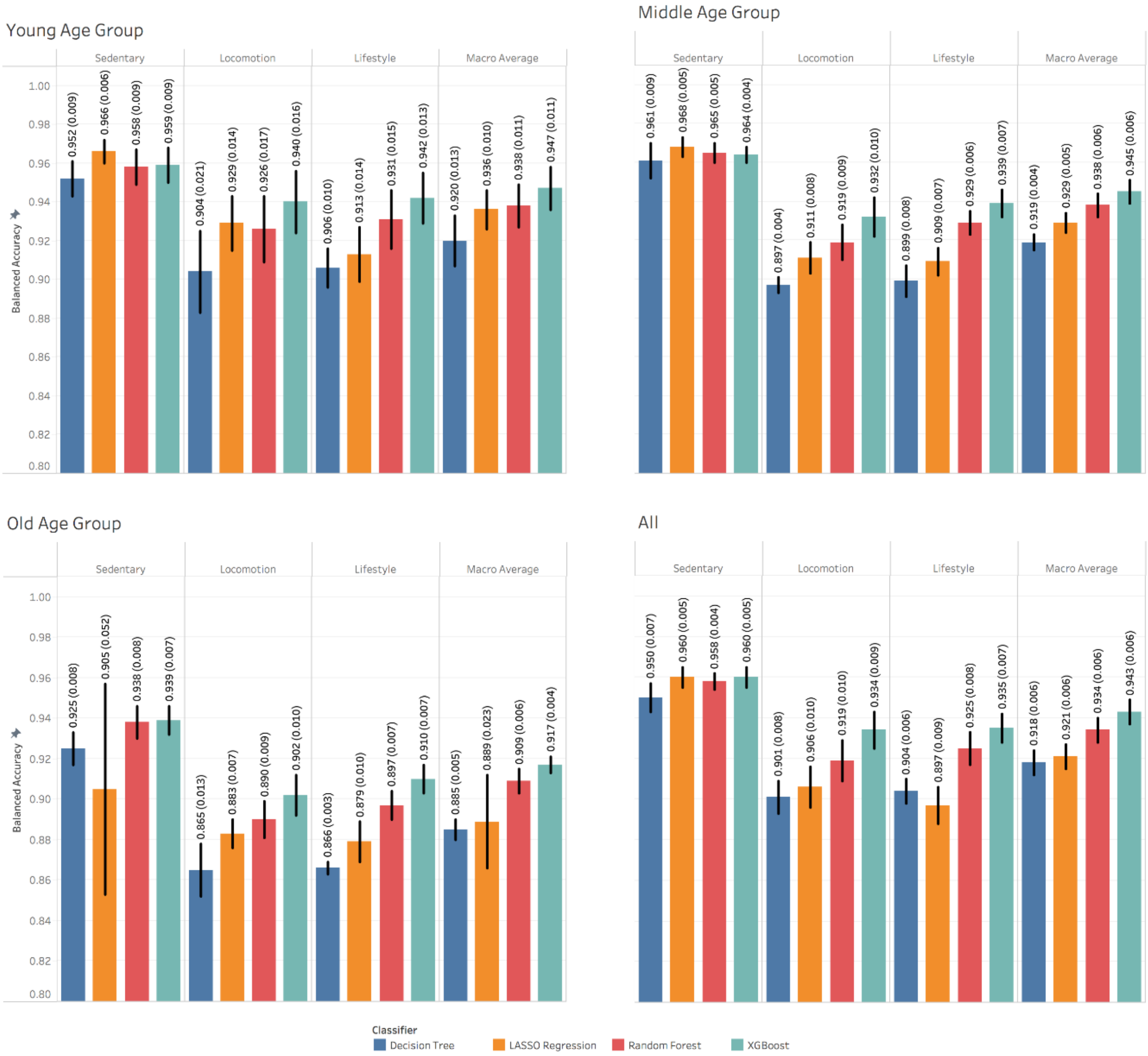

Figure S2. The balanced accuracy of recognizing physical activity type. Each value is the mean and standard deviation of the 5-fold nested cross validation.

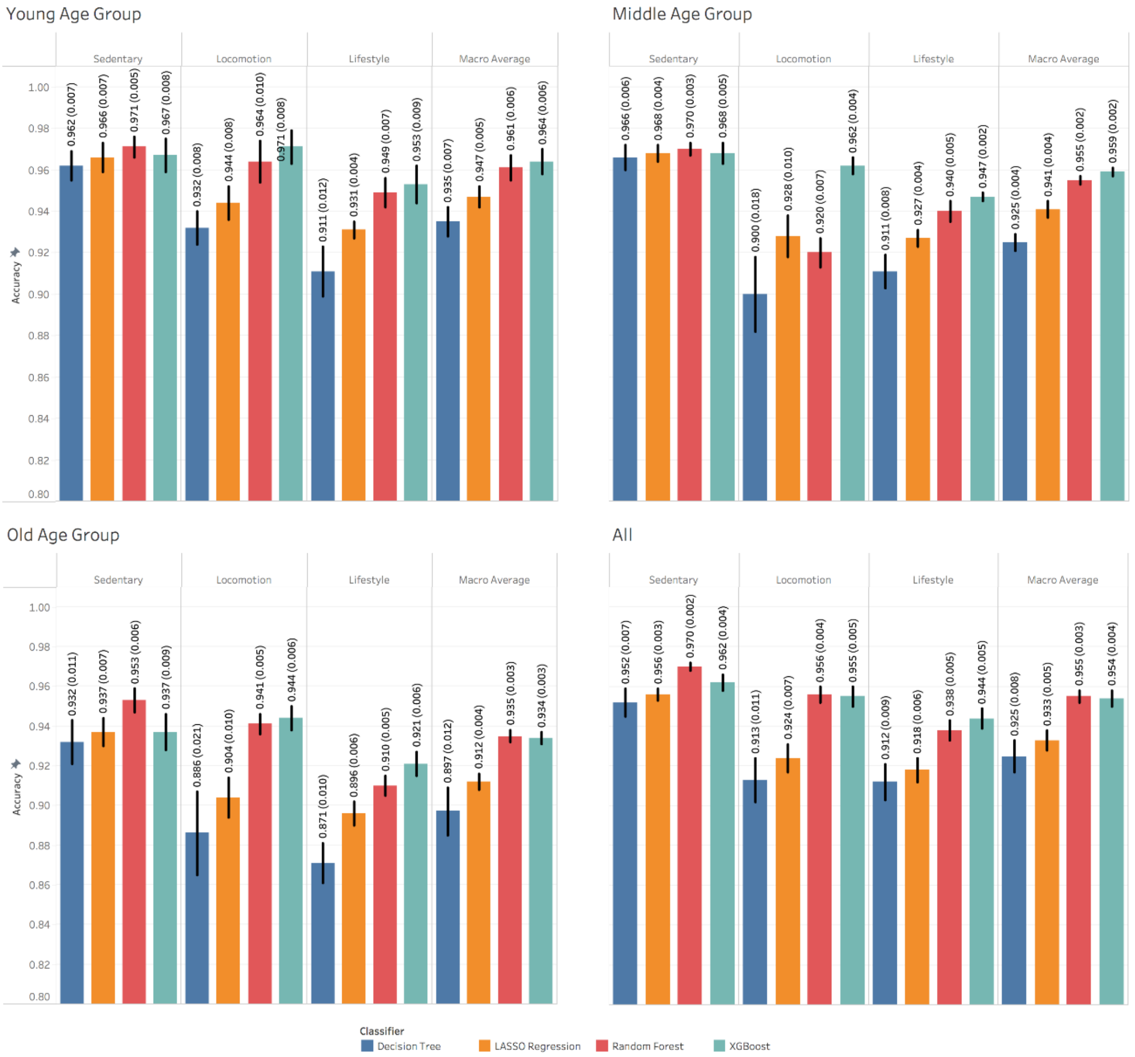

Figure S3. The accuracy of recognizing physical activity type. Each value is the mean and standard deviation of the 5-fold nested cross validation.

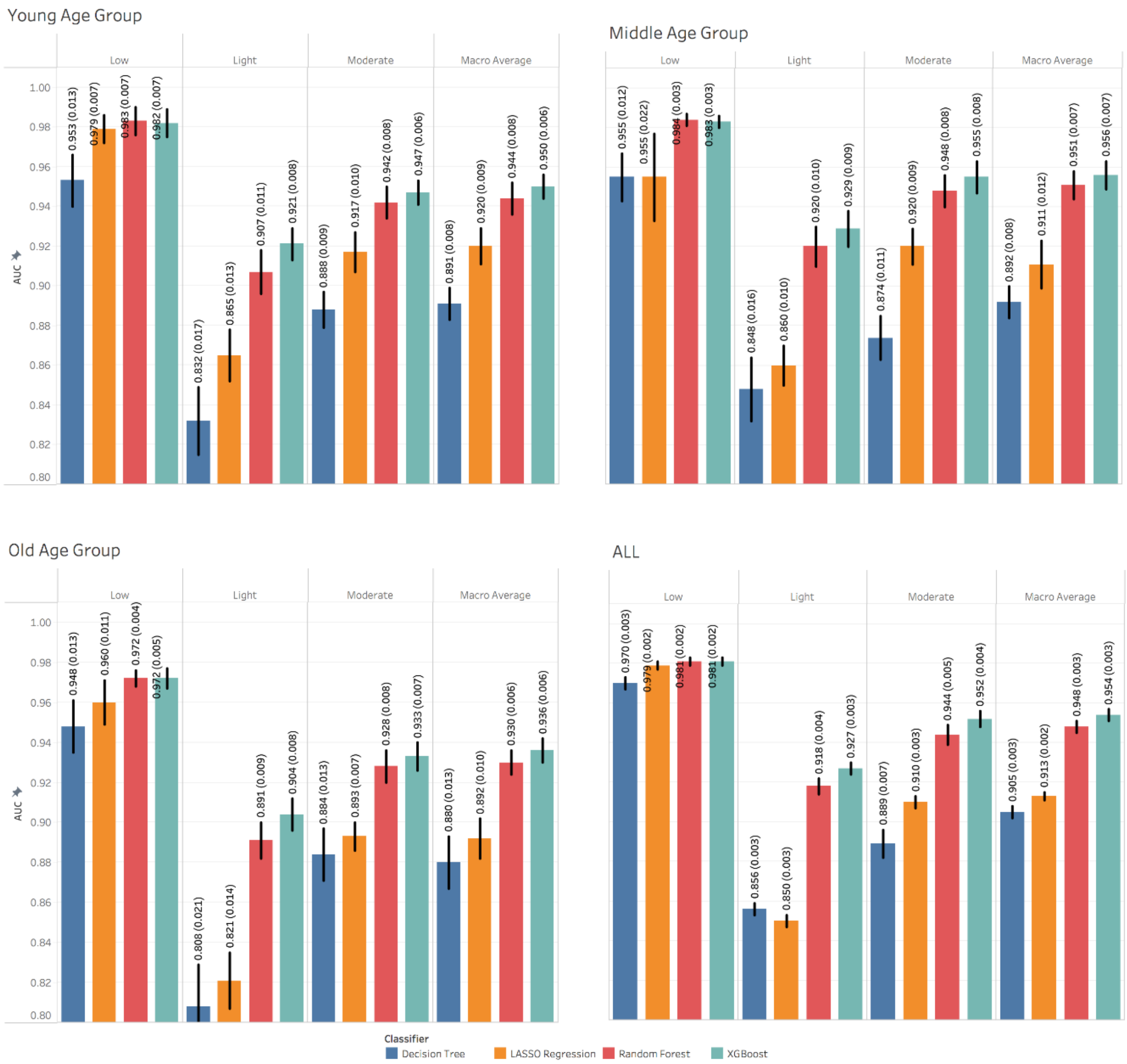

Figure S4. The receiver operating characteristic–area under the curve of recognizing physical activity intensity. Each value is the mean and standard deviation of the 5-fold nested cross validation.

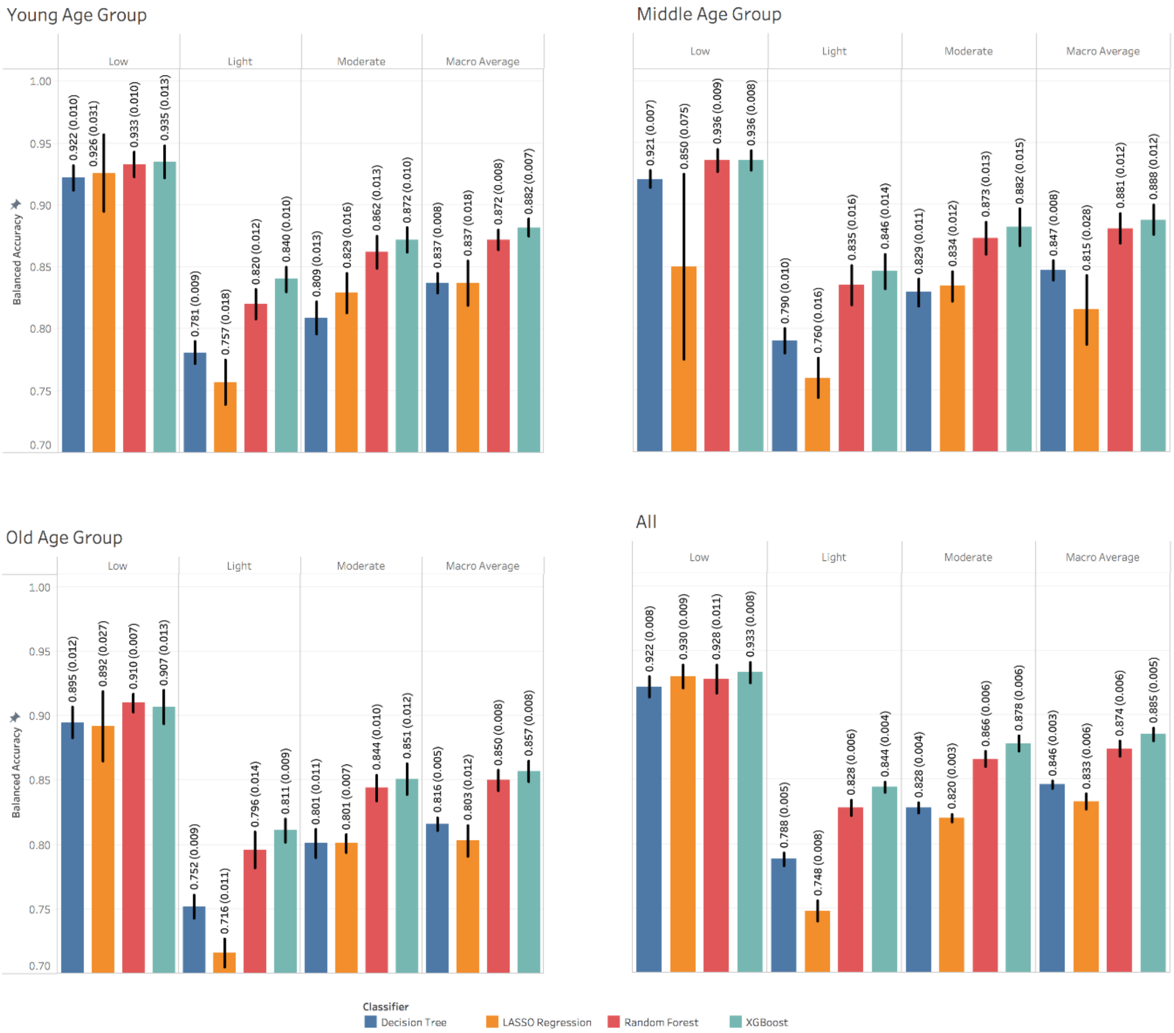

Figure S5. The balanced accuracy of recognizing physical activity intensity. Each value is the mean and standard deviation of the 5-fold nested cross validation.

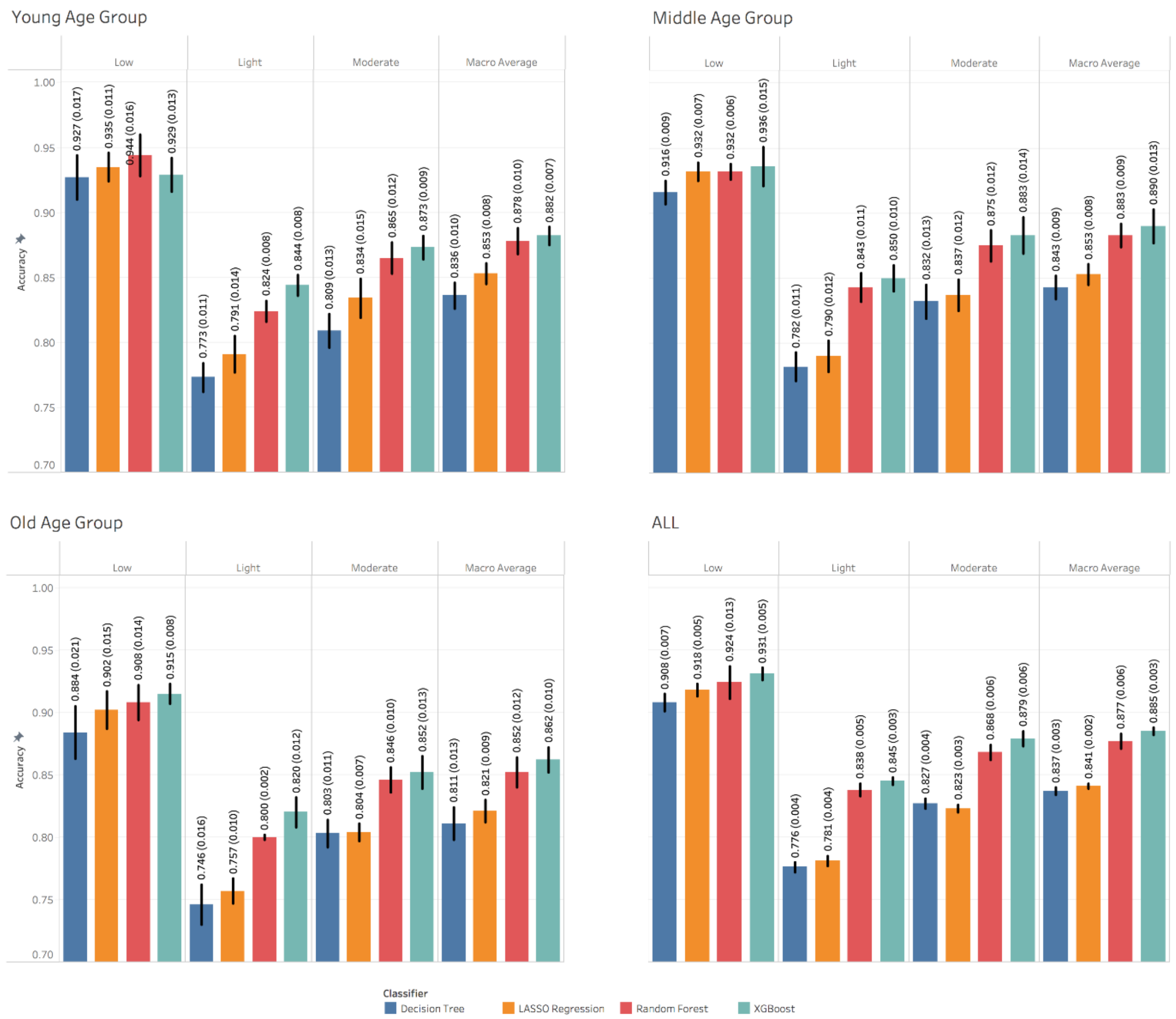

Figure S6. The accuracy of recognizing physical activity intensity. Each value is the mean and standard deviation of the 5-fold nested cross validation.

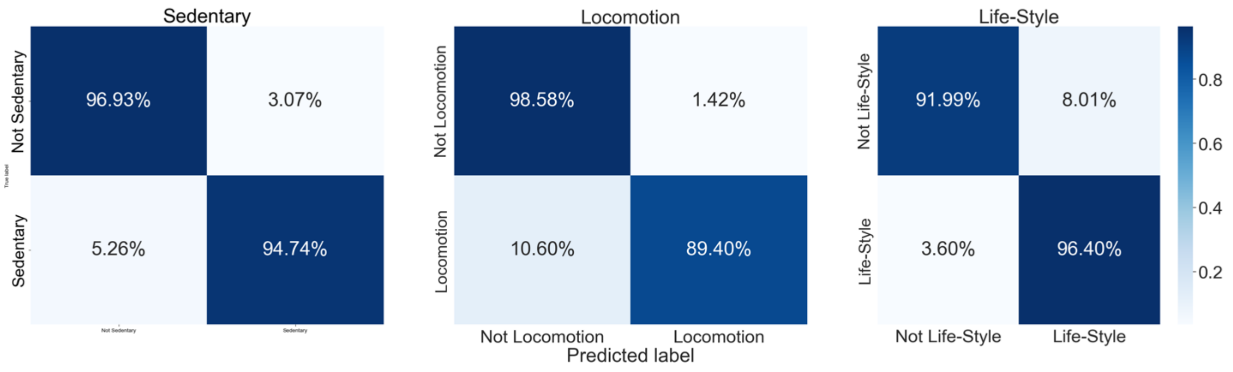

Figure S7. Confusion matrix of recognizing physical activity type for young age group.

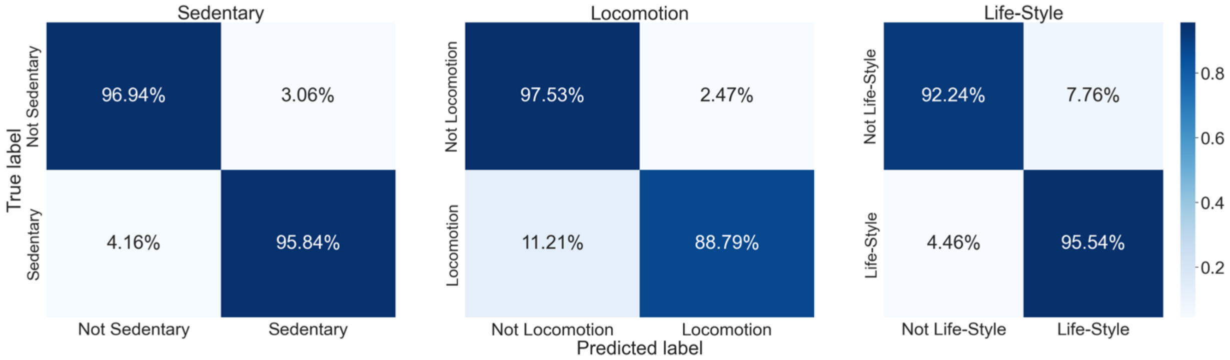

Figure S8. Confusion matrix of recognizing physical activity type for middle age group.

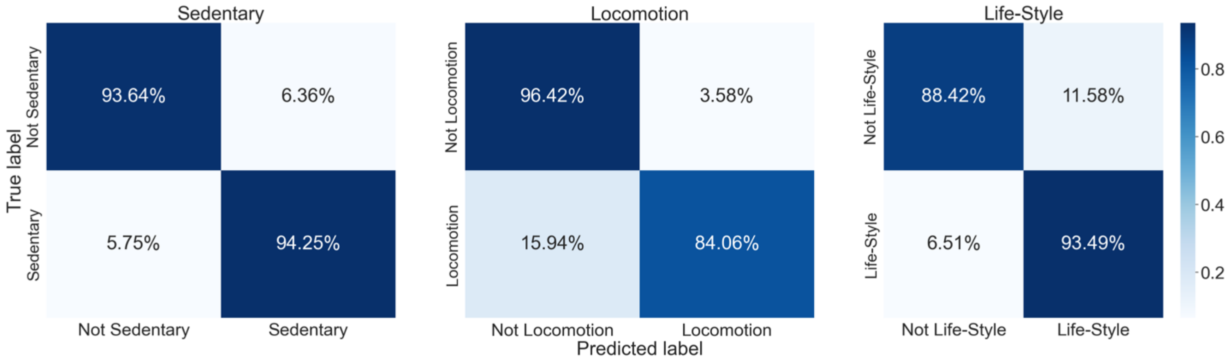

Figure S9. Confusion matrix of recognizing physical activity type for old age group.

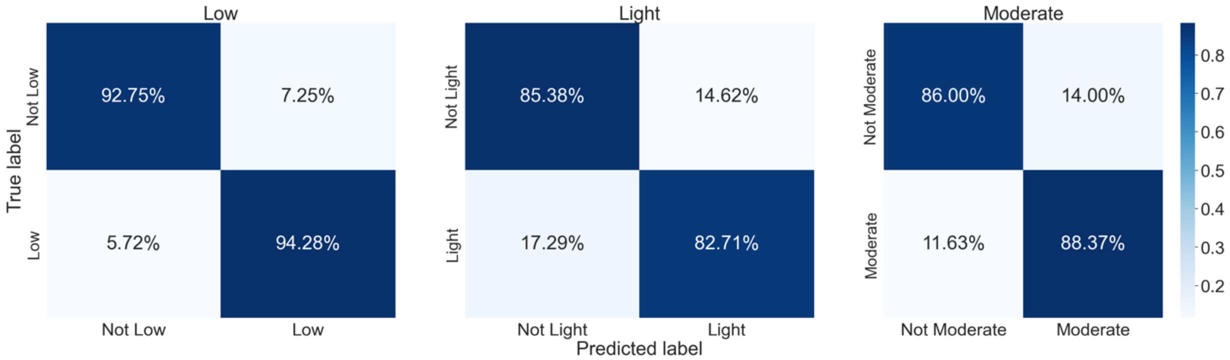

Figure S10. Confusion matrix of recognizing physical activity intensity for young age group.

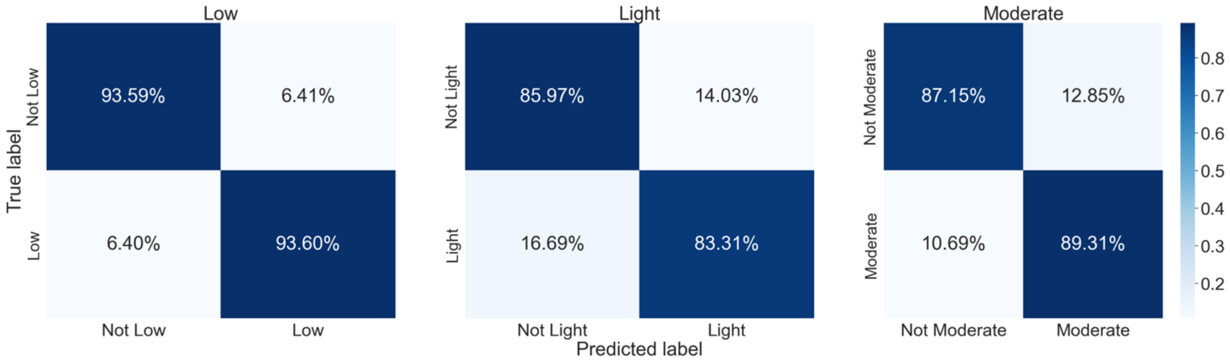

Figure S11. Confusion matrix of recognizing physical activity intensity for middle age group.

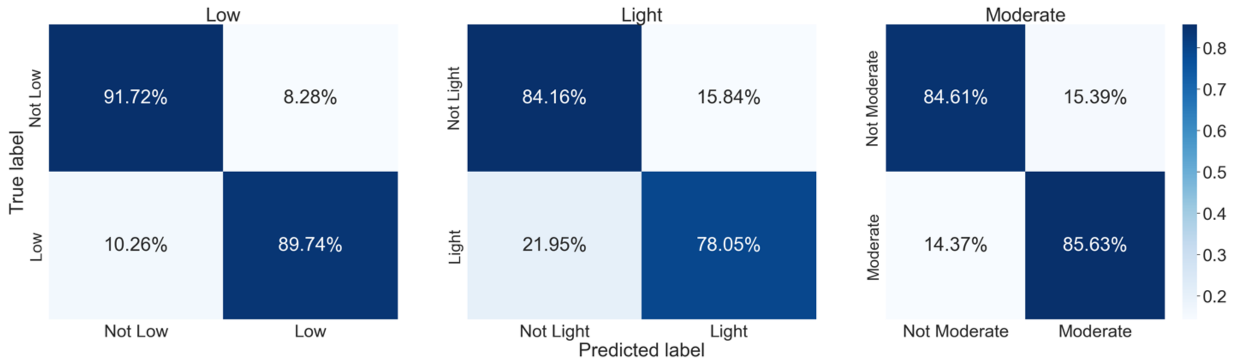

Figure S12. Confusion matrix of recognizing physical activity intensity for old age group.

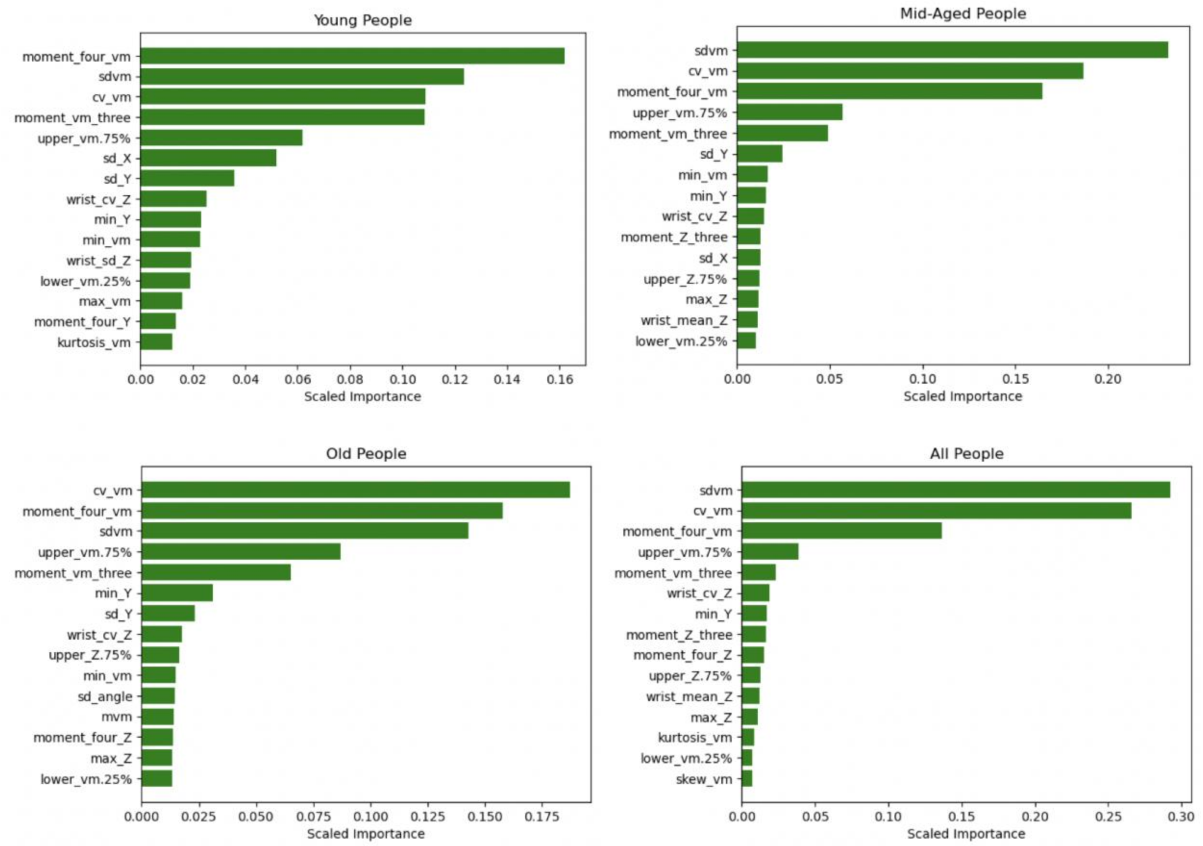

Figure S13. Feature importance for recognizing sedentary activities across age groups.

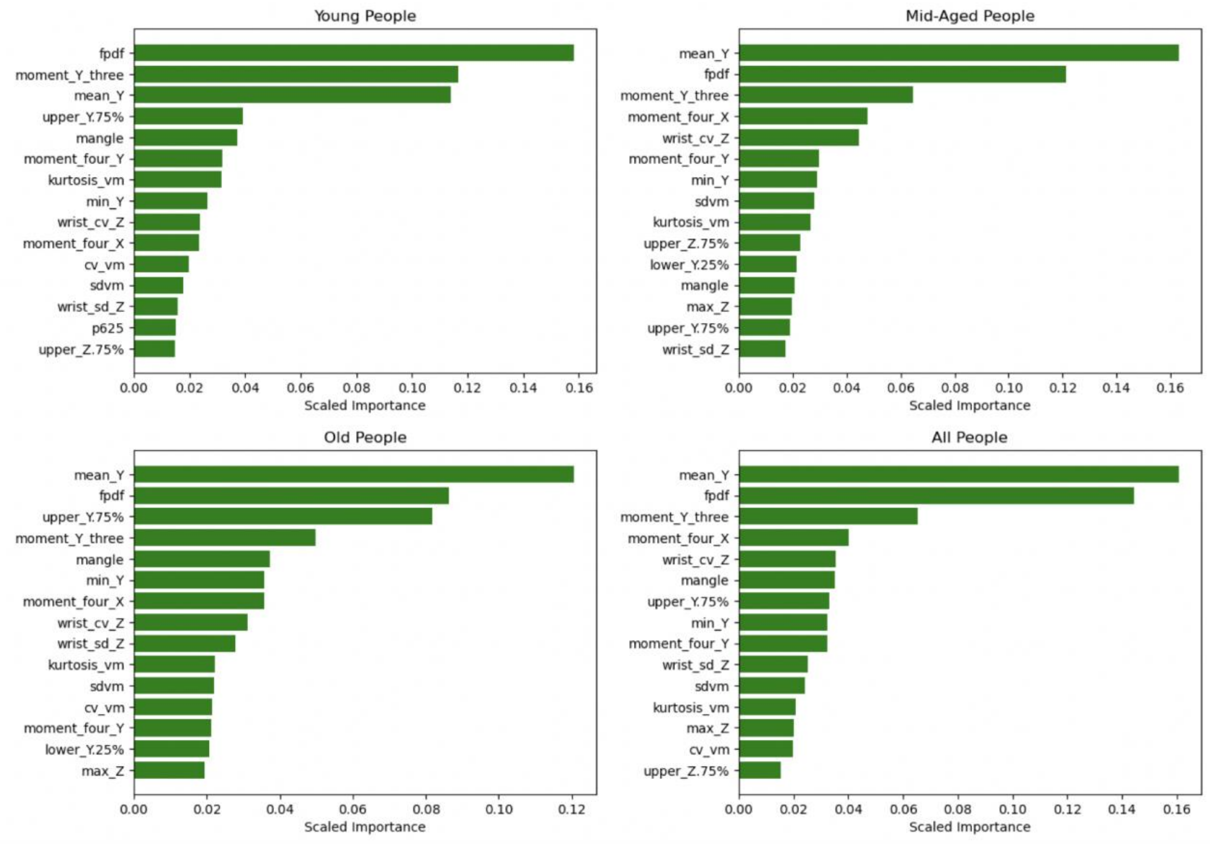

Figure S14. Feature importance for recognizing locomotion activities across age groups.

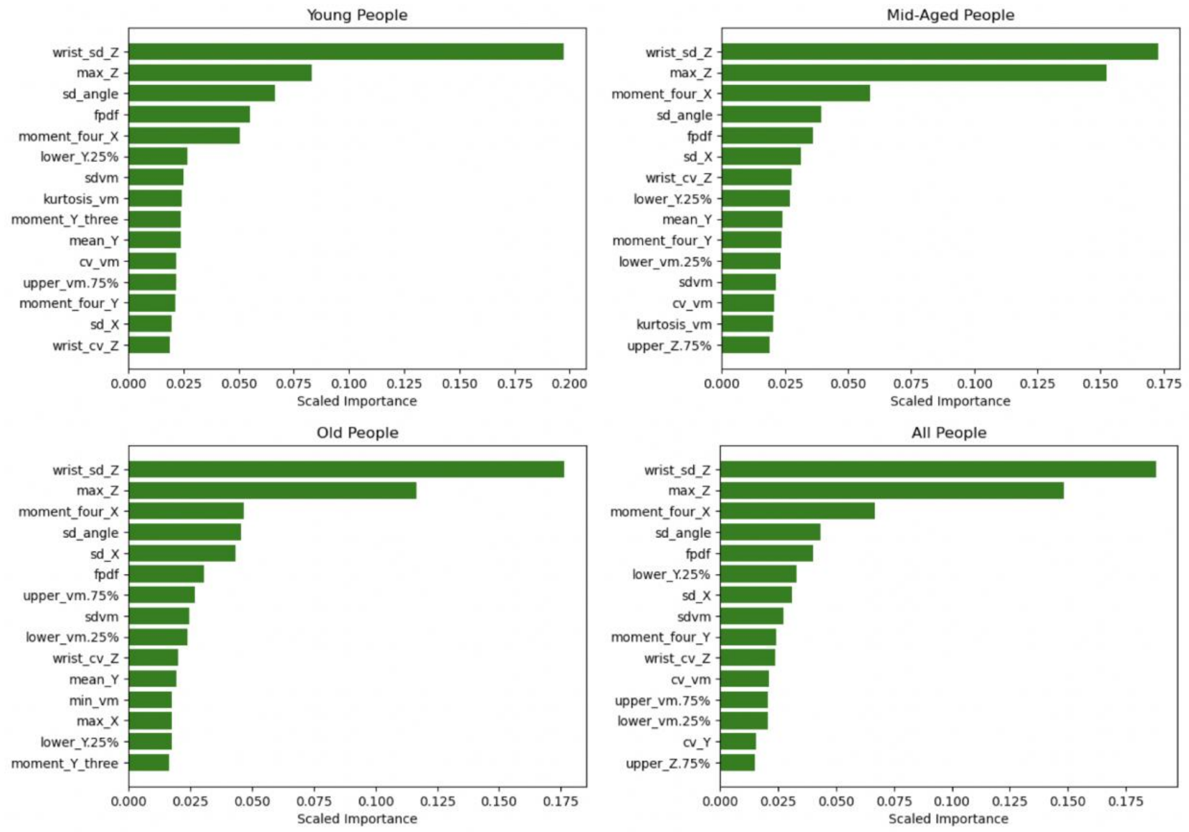

Figure S15. Feature importance for recognizing lifestyle activities across age groups.

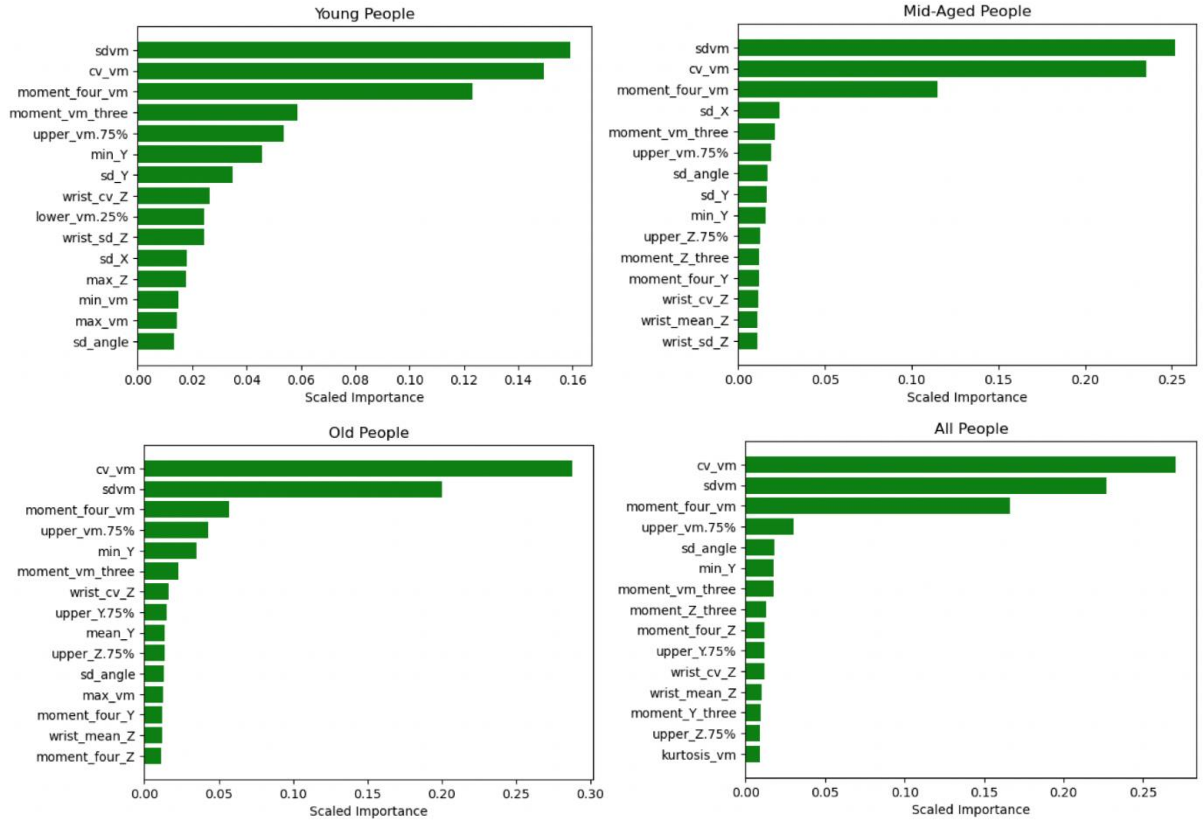

Figure S16. Feature importance for recognizing low intensity across age groups.

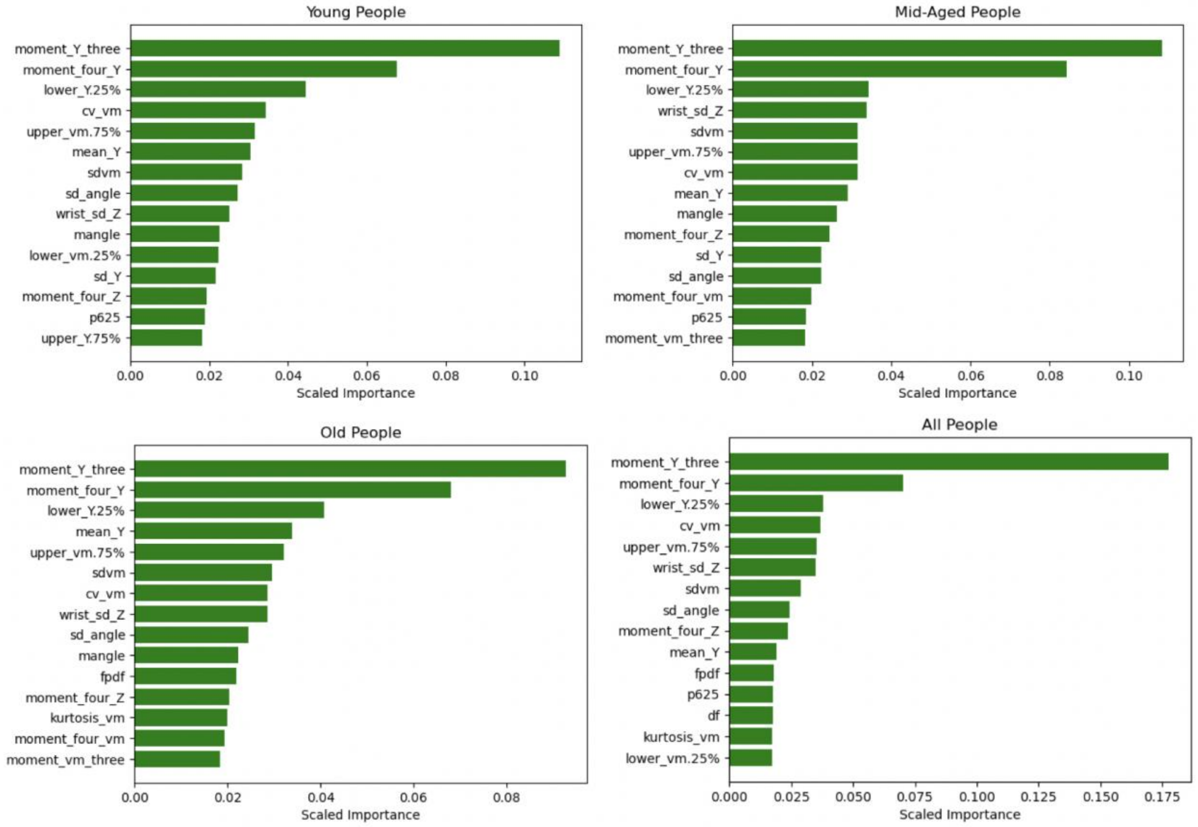

Figure S17. Feature importance for recognizing light intensity across age groups.

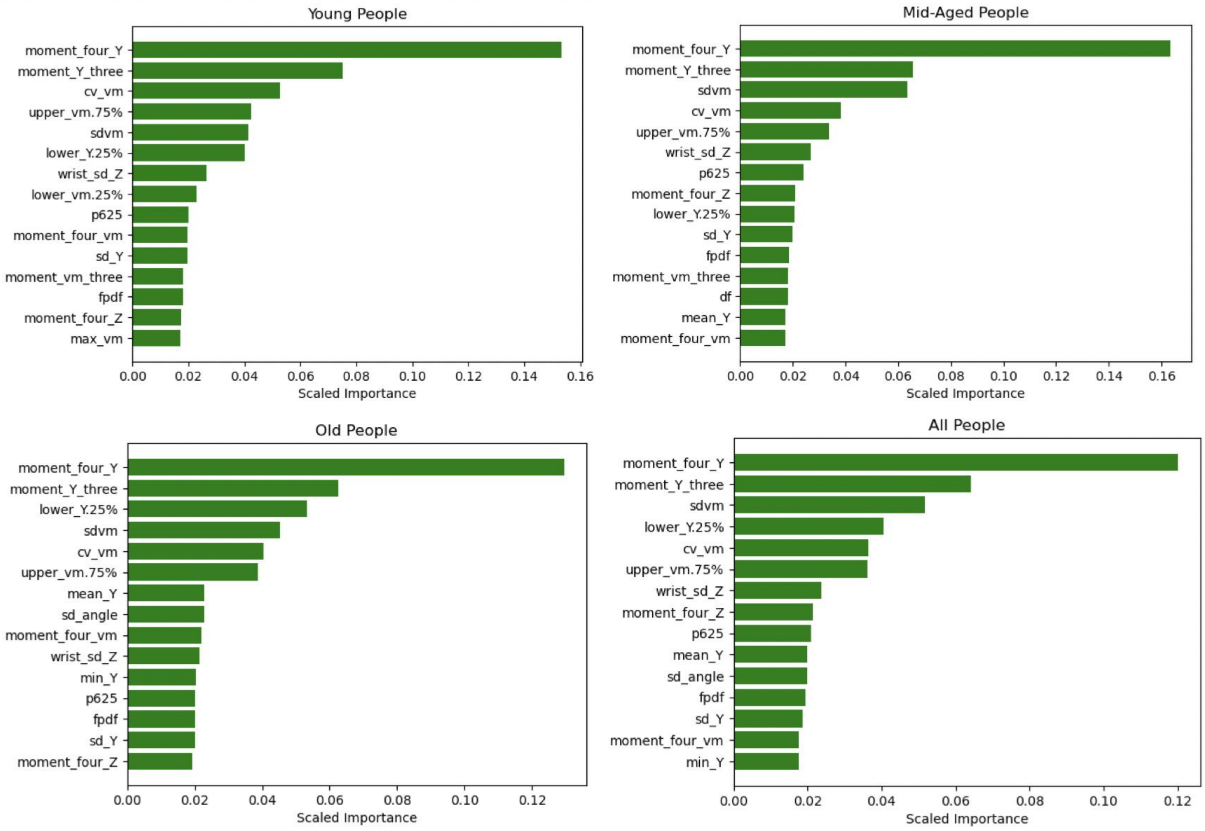

Figure S18. Feature importance for recognizing moderate intensity across age groups.

Table S2. Comparison with relevant work in the literature. The listed studies collected data from the wrist position for physical activity type classification. Classification performance is accuracy unless otherwise mentioned. For the purpose of comparison, we calculated the average accuracy for the physical activity type classification. N is the number of participants; Num is number; ML is machine learning; RMSE is the root mean square error; RF is random forest; SVM is support vector machine; and RLR is regularized logistic regression.

**Reference**	**N**	**Age**	**Num. Activities**	**Num. Categories**	**ML Method**	**Classification Performance**	**RMSE**
Our work	253	61.7 ± 17.7	33	3	RF	95.4% ± 0.004	0.898 ± 0.048
Mannini et al. [33]	33	18–75	26	4	SVM	84.7%	NA
Zhang et al. [56]	60	49.4 ± 6.5	10–12	4	DT	97.0%	NA
Ellis et al. [49]	40	35.8 ± 12.1	8	4 and 8	RF	87.5% and 80.2%	1.00
Trost et al. [57]	52	13.7 ± 3.1	12	7	RLR	88.4%	NA
Staudenmayer et al. [8]	20	24.1 ± 4.5	19	2 and 3	RF	96–99% and 76%	1.21
Ahmadi et al. [19]	31	4.0 ± 0.9	5	5	RF	F1 = 80.6	NA
Davoudi et al. [39]	40	55.2 ± 17.8	15	2	Multiple	98–100%	0.71

Studies that did not measure oxygen consumption were labeled as “not applicable” (NA).
